# Strategy for Cytoplasmic Delivery Using Inorganic Particles

**DOI:** 10.1007/s11095-022-03178-1

**Published:** 2022-02-02

**Authors:** Zhi Ping Gordon Xu

**Affiliations:** Australian Institute for Bioengineering and Nanotechnology, The University of Queensland, Brisbane, Queensland 4072 Australia

**Keywords:** anti-acidification, cellular delivery, endosome escape, lysosome escape, norganic nanomaterials

## Abstract

Endosome escape is a key process for intracellular uptake of intact biomolecules and therapeutics, such as nucleic acids. Lysosome escape is a more common pathway during endocytosis, while some biomolecular, organic and inorganic materials are found to enhance the endosome escape, and several mechanisms have been proposed accordingly. Specifically, some inorganic nanomaterials show their unique mechanisms of action for enhanced endosome escape, including salt osmotic effect and gas blast effect. These inorganic nanomaterials are basically weakly alkaline and are naturally featured with the anti-acidification capacity, with limited solubility in neutral solutions. This review paper has briefly presented the strategies in the design of inorganic nanoparticle-based cellular delivery vehicles with endosome escapability and discussed a few typical inorganic nanomaterials that are currently widely examined for delivery purpose. A brief summary and prospect for this kind of inorganic nanomaterials are provided.

## Introduction

Nanoparticles have been now extensively developed for delivery of many different types of therapeutics (1). As is well known, nanoparticles are generally able to load and controllably release cargoes, deliver in some targeted ways, and provide some protection of cargoes during *in vitro* and *in vivo* delivery (2, 3). Applications of specific therapeutics often require their delivery to specific intracellular compartments because their sites of biological actions are different. For example, doxorubicin and pDNA have to be delivered to the cell nucleus (4), and siRNA and mRNA just require delivery to the cytoplasm (5). These therapeutics must be intact prior to their interactions with the target molecules or organelles at the site of action.

**Fig. 1:**
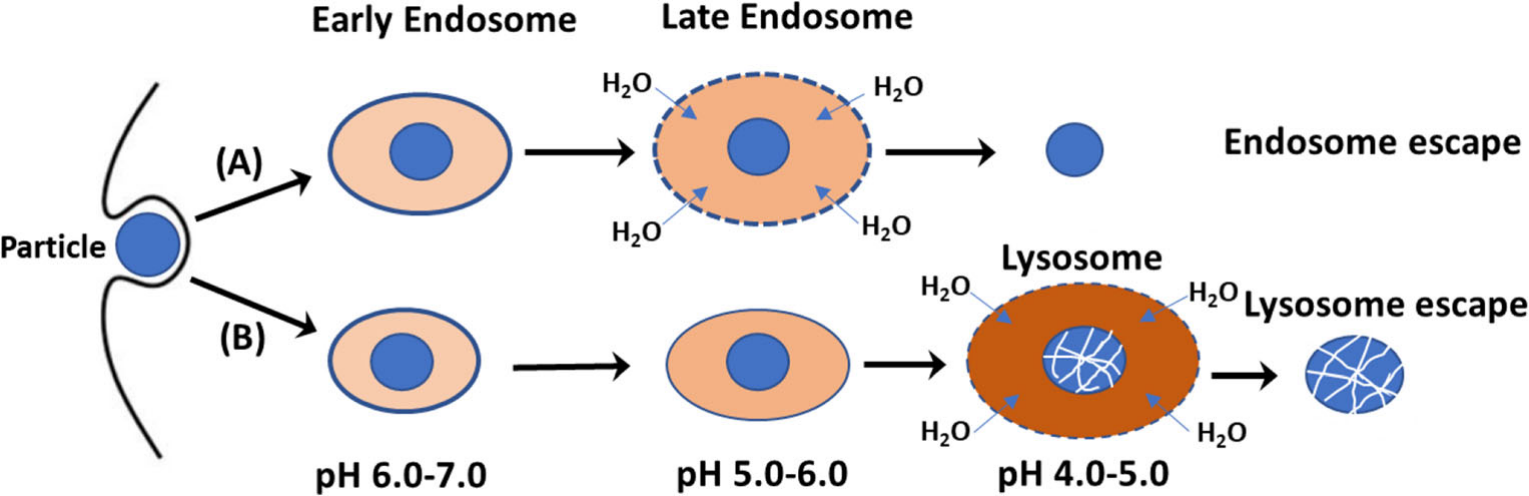
Two endocytosis pathways. (**A**): endosome escape; (**B**): lysosome escape. The darker color of vesicles represents the lower pH and the larger size means their expansion due to the water influx induced by the osmotic pressure.

A big challenge for achieving efficient intracellular delivery of these intact bioactives is how to efficiently escape from endosomes, not from lysosomes for most nanoparticles (2, 3). In general, nanoparticles are internalized by cells through the endocytosis pathway (Fig. 1). Endocytosis normally involves formation of an endocytic vesicle as an early endosomal compartment, maturation into a late endosome. The late endosome is then fused as a lysosome where nanoparticles and cargo are liable to be degraded by various nucleases, and followed by the escape to the cytosol. During endocytosis, the pH decreases from physiological pH 7.4 in the endocytic vesicle to pH 6.0–7.0 in the early endosome, pH 5.0–6.0 in the late endosome, and pH 4.0–5.0 in the lysosome (6, 7). This pathway (lysosome escape, Fig. 1B) may be associated with degradation of internalized nanoparticles/cargos, and may fail to deliver intact therapeutics (particularly proteins and genes) to the sites of action. Thus, in order to efficiently deliver intact therapeutics to the cytoplasm, the endosome escape is necessary for the intact bioactive delivery (Fig. 1A). This review will focus on the recent progresses using specifical inorganic nanoparticles for the successful cytoplasmic delivery of intact therapeutics via the endosome escape pathway.

## Basic Mechanisms of Endosome Escape of Delivery Systems

It is important to deeply understand the mechanisms governing endosomal escape for rational design of efficient cytoplasmic delivery nanoplatforms. Currently, several endosomal escape mechanisms are proposed, including proton sponge effect, reverse (salt) osmotic effect, membrane fusion and rupture, and other ways (2, 3, 5). These mechanisms are proposed for the (bio)organic delivery systems (such as polymers, proteins, peptides and lipids), while some mechanisms are also applied to inorganic nanoparticle systems. Moreover, some (bio)organic molecules are conjugated to inorganic nanoparticle systems to enable the hybrid system to escape from the endosome. Thus, the following summary is based on the (bio)organic delivery systems, but also related to the inorganic nanoparticle platforms.

### Proton Sponge Effect

The proton sponge effect is proposed to explain the nucleic acid delivery with polymeric carriers that are positively charged in physiological solutions, including polyethyleneimine (PEI) and polyamidoamine (8). These polymers are thought to buffer the protons pumped into endosomes and/or lysosomes as a proton sponge as the amine groups prevent endosomal acidification by neutralizing H^+^ ions pumped by ATPase. As consequence, chloride ions are simultaneously transferred into the endosome via the ion channel to keep the charge balanced, and thus the generated osmotic pressure causes the endosome swelling, leading to endosome rupture and releasing polymer-nucleic acid complexes into the cytosol. This hypothesis is often debated by many researchers (9). The author believes that the proton sponge effect should be very weak when the pH is changed from 7.4 to 5.0 or even 4.0, which could be evaluated through the following equation:

1 $$ \mathrm{R}-\mathrm{N}{{\mathrm{H}}_3}^{+}\kern0.75em \rightarrow \kern1em \mathrm{R}-\mathrm{N}{\mathrm{H}}_2+{\mathrm{H}}^{+}\kern2em \mathrm{supposing}\ \mathrm{pKa}=9.0 $$

There is 97.5% of amine groups protonated (R-NH_3_^+^) in pH 7.4 buffer, as simply estimated using [R-NH_2_]/[R-NH_3_^+^] = 10^pH-pKa^. This estimation means that only 2.5% of amine groups (R-NH_2_) in the polymer is available to buffer and capture the incoming protons. This limited proton sponge capacity may not help capture many H^+^ ions so as to cause the enough osmotic pressure and lead the rupture of endosome, achieving the endosome escape of the bioactive to the cytoplasm. Based on this analysis, the author has doubted the feasibility of H^+^ sponge effect for PEI based systems. Instead, some inorganic nanomaterials exhibit “H^+^ sponge” in some way to capture pumped-in H^+^ ions and achieve endosomal escape, as discussed in the section of “typical inorganic nanomaterials”.

### Reverse Osmotic Effect

If the amine group in the polymer has a pKa of 6–7 (such as imidazole group in histidine), then polymeric nanoparticles may have a strong proton sponge capacity when the pH changes from 7.4 to 5.0:

2 $$ \mathrm{R}1-\mathrm{N}{{\mathrm{H}}_2}^{+}-\mathrm{R}2\kern0.75em \rightarrow \kern1em \mathrm{R}1-\mathrm{N}\mathrm{H}-\mathrm{R}2+{\mathrm{H}}^{+}\kern0.75em \mathrm{supposing}\ \mathrm{pKa}=6.0 $$

As estimated similarly, there is only 3.8% of imidazole groups protonated (R1-NH_2_^+^-R2) in pH 7.4 solution, which means that over 90% of imidazole groups (R1-NH-R2) can be used to buffer the incoming protons in the endosome. Associated with the proton buffer is the influx of chloride counterions for the charge balance in the endosome, which causes a further influx of water molecules due to the osmotic pressure, increasing the endosome volume and eventually breaking out the endosome to successfully deliver the intact bioactive to the cytoplasm.

This reverse osmotic effect is observed for several inorganic nanomaterials, such as layered double hydroxide (10, 11), calcium phosphate (12–14) and calcium carbonate (15), which are sensitive to the acidity and semi-dissolvable in acidic solutions, as extensively discussed shortly.

### Membrane Fusion and Rupture

A good example is virus whose membrane is able to fuse with the endosomal membrane to allow the virus to diffuse into the cytoplasm. Based on this phenomenon, some liposomes are specifically designed to undergo the endosomal escape through fusion with the endosome membrane. This fusion normally involves the cell-penetrating peptides (CPPs) that are conjugated on the liposome surface (16). CPPs are polycationic or amphipathic peptides with relatively abundant positively charged amino acids or an alternating pattern of polar, charged amino acids and non-polar, hydrophobic amino acids (17). These peptides can cross the cell membrane but do not damage the membrane. In general, membrane fusion should completely deliver the bioactives to the cytoplasm. However, the delivery efficacy of CPP-modified lipid nanoparticles is not high, which suggests that other mechanism (such as lysosomal escape) may occur in parallel.

Membrane destabilization and disruption are proposed to explain the endosomal escape of some specific polymeric delivery systems. It is hypothesized that some specific groups of polymers can bind with the endosomal membrane via electrostatic and/or hydrophobic interactions, which destabilizes and ruptures the local membrane to release the cargo to cytoplasm (18). This is somewhat similar to the mechanism of pore formation in the endosome membrane (19).

It is worth mentioning that this endosomal escape pathway is related to specific interactions between (bio)organic systems and the cell membrane. These interactions seem not to be possible for inorganic nanoparticle systems, while conjugation of (bio)organic molecules to inorganic nanoparticles enables the hybrid system to achieve endosomal escape as well, as discussed shortly.

### Other Mechanisms

Some other pathways are proposed to explain the endosome escape, including polymeric nanoparticle (hydrogel) swelling, pore formation on the endosome membrane (19), vesicle budding and collapse, as well as gas blast effect (20–22), where the last mechanism is mainly related to inorganic nanomaterials. As most mechanisms are related the specific materials and not widely applied, deep discussion is not provided.

## Strategies for Endosome Escape of Inorganic Nanomaterials

### Inorganic Nanomaterials with Inherent Endosome Escapability

As mentioned previously, the main feature of endosome maturation is acidification through the ATPase to transport H^+^ ions from the cytosol. Based on this feature, several inorganic nanomaterials with inherent anti-acidification capacity are extensively examined as delivery systems, as reviewed below. These inorganic nanomaterials are weakly alkaline materials with poor solubility in water, including some hydroxides and mixed hydroxides, phosphates, carbonates and their mixtures. These nanomaterials (simply expressed as M_x_A_y_) are able to neutralize H^+^ ions pumped into the endosome through ATPase, as exemplified in the following equation:

3 $$ {\mathrm{M}}_{\mathrm{x}}{\mathrm{A}}_{\mathrm{y}}+\mathrm{y}{\mathrm{H}}^{+}\rightarrow \kern0.75em \mathrm{x}{\mathrm{M}}^{\mathrm{m}+}+\mathrm{y}\mathrm{H}{\mathrm{A}}^{1-\mathrm{mx}/\mathrm{y}} $$

**Fig. 2:**
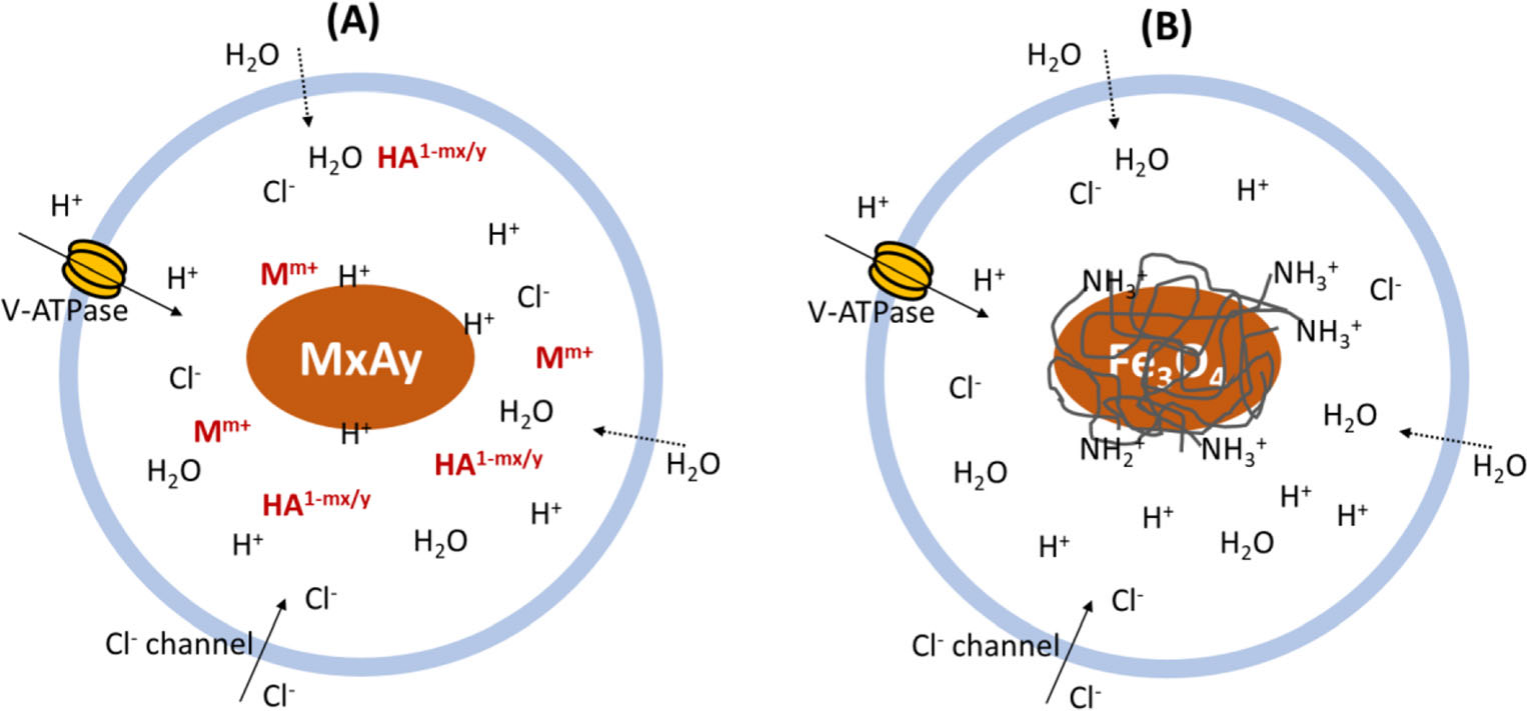
Two strategies for developing inorganic nanoparticle-based delivery systems with endosome escapability. (**A**) using the inherent weak alkalinity of inorganic nanoparticles to neutralize the transported H^+^ ions to generate ‘salt osmotic effect’; (**B**) using the buffer capacity of (bio)polymers that are coated or conjugated onto the inorganic nanoparticles (Fe_3_O_4_) to generate ‘proton sponge effect’. Similarly, CPP peptide modification can be conjugated onto inorganic nanoparticles to cause the membrane fusion (not shown here).

In such a way, the poorly soluble inorganic nanoparticles are triggered by the acidity to slowly dissolve, releasing metal ions M^m+^ and anions HA^1-mx/y^ (Fig. 2A). The increased ionic strength (as well as associated Cl^−^) then generates a considerable osmotic pressure in the endosome, which causes an influx of cytosol water molecules into the endosome, swells and finally bursts the endosome (salt osmotic effect) (10, 11, 13). This endocytosis pathway thus avoids lysosome degradation and achieves cytoplasmic delivery of the nanoparticle-bioactive complexes.

Note that many inorganic nanoparticles, such as various SiO_2_-based nanoparticles, carbon-based nanomaterials (such as nanotubes, carbon dots, and graphene sheets), metal nanoparticles (such as Au, Ag, and Cu nanomaterials) and metal oxides (such as Fe_3_O_4_ and Al_2_O_3_), have limited capacity to neutralize incoming H^+^ and dissolve in the endosome, and very probably enter the lysosomal pathway. However, the nanomaterials can be modified in some ways and change to the endosome pathway, as discussed subsequently.

### Modification of Inorganic Nanomaterials for Endosome Escape

These inorganic nanoparticles, such as gold (AuNP) and iron oxide (IONP) do not have the inherent anti-acidification property but can be modified into hybrid delivery systems for endosome escape (23). As mentioned previously, (bio)polymers with specific pH-sensitive groups and CPPs can also help the delivery system escape from the endosome via mechanisms such as so-called proton sponge effect, reverse osmotic effect, and membrane fusion and rupture (Fig. 2B). For example, colloidally stable IONP-PEI-DNA beads enhanced endosomal escape and effectively transfected COS cells (cell lines) (24). IONPs were also coated with transactivator of transcription (TAT) peptide (a CPP peptide) to escape from the endosome very effectively (25). In another example, TAT peptide conjugated onto FITC-IONPs enhanced the cytosolic delivery while most blank nanoparticles were trapped within the endosome/lysosome (26).

## Typical Inorganic Nanomaterials with Inherent Property for Endosome Escape

Several inorganic nanoparticles are designed to deliver therapeutics through the endosome pathway (Fig. 1A). Here their inherent anti-acidification property naturally facilitates the endosome escape, including semi-soluble/poorly soluble hydroxides (such as Mg(OH)_2_ and Zn(OH)_2_), layered double hydroxides (MgAl-mixed hydroxides), some carbonates and phosphates. The following section will focus on the extensively examined inorganic nanoparticle delivery systems, i.e. layered double hydroxide (LDH), calcium phosphate (CaP) and calcium carbonate (CaC), as well as their hybrid (inorganic/organic and inorganic/inorganic) nanoparticles.

### Layered Double Hydroxide

Layered double hydroxide (LDH), also known as anionic clay, is a mixed hydroxide (27, 28). The general formula of LDH is [M_1-x_^2+^M_x_^3+^(OH)_2_]^x+^(A^n-^)_x/n_·mH_2_O (29), where M^2+^ represents divalent metal cations (e.g. Mg^2+^, Fe^2+^ and Zn^2+^), M^3+^ trivalent metal cations (e.g. Al^3+^ and Fe^3+^), A^n-^ anions (e.g. Cl^−^ and CO_3_^2−^) and x denotes the molar fraction of trivalent in all metal cations. Different from silica-base layered materials, typical LDH (such as MgAl-LDH) has positively charged hydroxide layers, which enable to carry a high amount of anionic therapeutics (e.g. DNA, siRNA, and negatively charged drugs) in the interlayer spacing and on the surface (30), and enhance the cellular uptake (10, 11, 31, 32) with good protection of the cargoes from enzymatic degradation (33). Many reports have shown that the unique plate-like structure and tailorable particle size of MgAl-LDH enable to efficiently interact and deliver bioactives into cells with excellent biocompatibility (34).

**Fig. 3:**
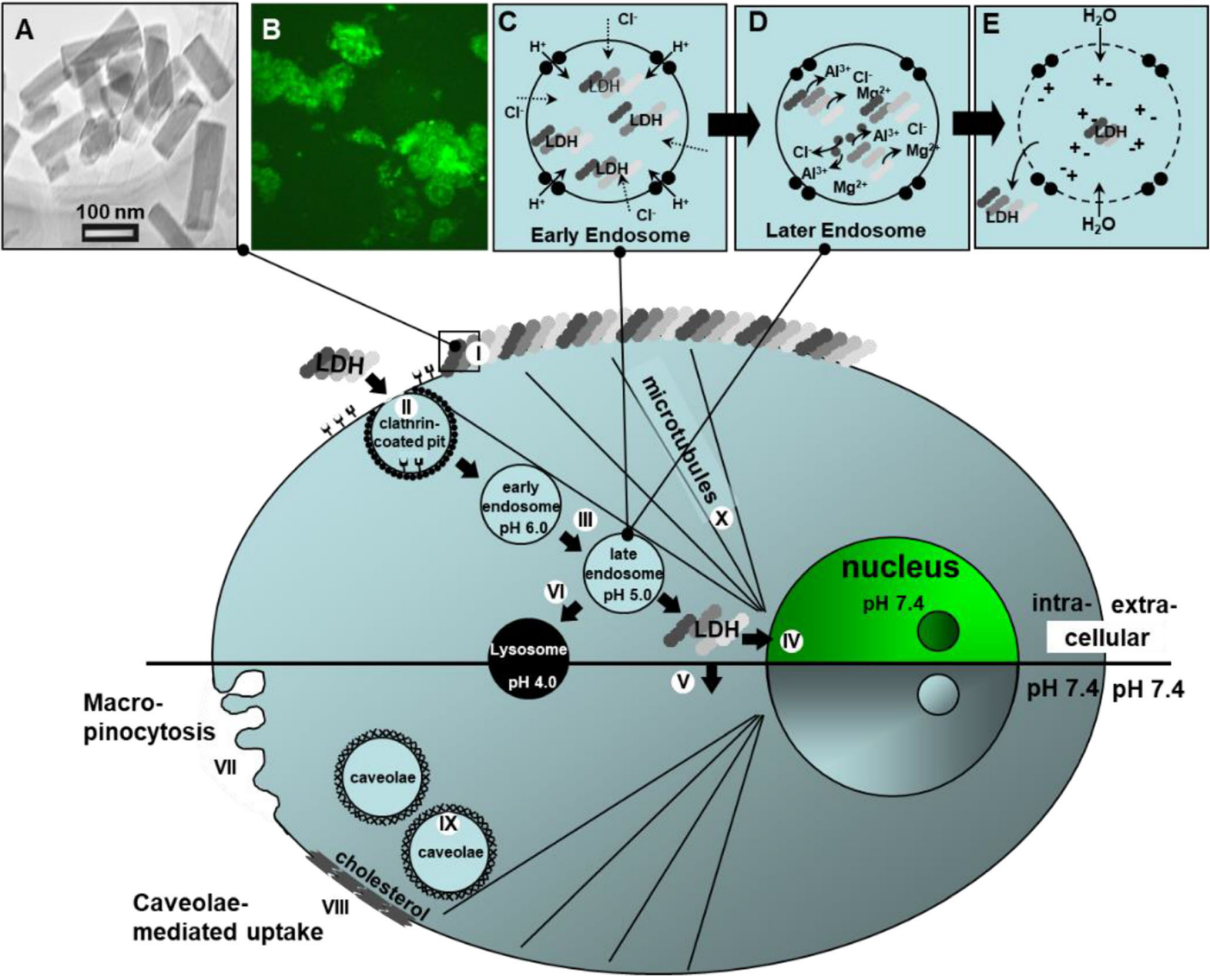
Endosomal escape mechanism of LDH. (**A**) TEM image of LDH-FITC nanoparticles; (**B**) CHO (Chinese hamster ovary) cells after internalization of LDH-FITC nanoparticles; (**C**) Pumping protons to acidify the endosome, which is followed by chloride ion influx; (**D**) LDH-FITC nanoparticles are acid-triggered to dissolve in the endosome; (**E**) Water molecule influx to the endosome due to the osmotic pressure, swelling and bursting the endosome to release LDH-FITC nanoparticles into cytosol. *Step I*: Adhesion of LDH-FITC nanoparticles to the cell membrane; *Step* II: Clathrin-mediated endocytosis; *Step III*: Endosomal acidification; *Step IV*: Nuclear localization of LDH-FITC; *Step V*: Distribution of LDH-FITC in cytosol; *Step VI*: Lysosomal pathway; *Step VII*: Proposed macropinocytosis. *Step VIII-IX*: Caveolae-mediated endocytosis; *Step X*: Microtubule directing LDH-FITC nanoparticles to the nucleus. Reproduced with permission (10) with slight modifications. Copyright 2008, Elsevier.

The LDH nanoparticles are internalized by cells via the clathrin-mediated endocytosis (Fig. 3). As the endosome is gradually acidified via pumped protons for proton-mediated dissolution of LDH-bioactive nanoparticles, the endosome pH gradually decreases, which leads to partial dissolution of LDH nanoparticles via the following neutralization reaction to buffer the acidity in the endosome:

4 $$ {\mathrm{Mg}}_2\mathrm{Al}{\left(\mathrm{OH}\right)}_6\mathrm{Cl}+\left(2\mathrm{m}-\mathrm{n}\right){\mathrm{H}}^{+}\rightarrow {\mathrm{Mg}}_{2-\mathrm{m}}{\mathrm{Al}}_{1-\mathrm{n}}{\left(\mathrm{OH}\right)}_{6-2\mathrm{m}-2\mathrm{n}}{\mathrm{Cl}}_{1-\mathrm{n}}+\mathrm{m}{\mathrm{Mg}}^{2+}+{\mathrm{nCl}}^{-}+\mathrm{nAl}{\left(\mathrm{OH}\right)}_3 $$

Such a neutralization keeps the endosome pH at around 6 and simultaneously increases the ionic strength (together with the influx of chloride ions via the ion channel), which leads water molecules to enter the endosome and swell the endosome, eventually bursting the membrane and deliver the residual LDH-cargo and released cargos into the cytoplasm (Fig. 3). Since LDH is weakly alkaline and sensitive to acidity, this inherent anti-acidification property helps the LDH-cargo complexes to escape from the endosome and avoid lysosomal biodegradation. This escape mechanism is also described as “salt osmotic effect” (35). The internalized LDH nanoparticles are mainly localized in the cytoplasm after the endosome escape. Note that the dissolved LDH is minimal, approximately <1% of internalized LDH nanoparticles.

**Fig. 4:**
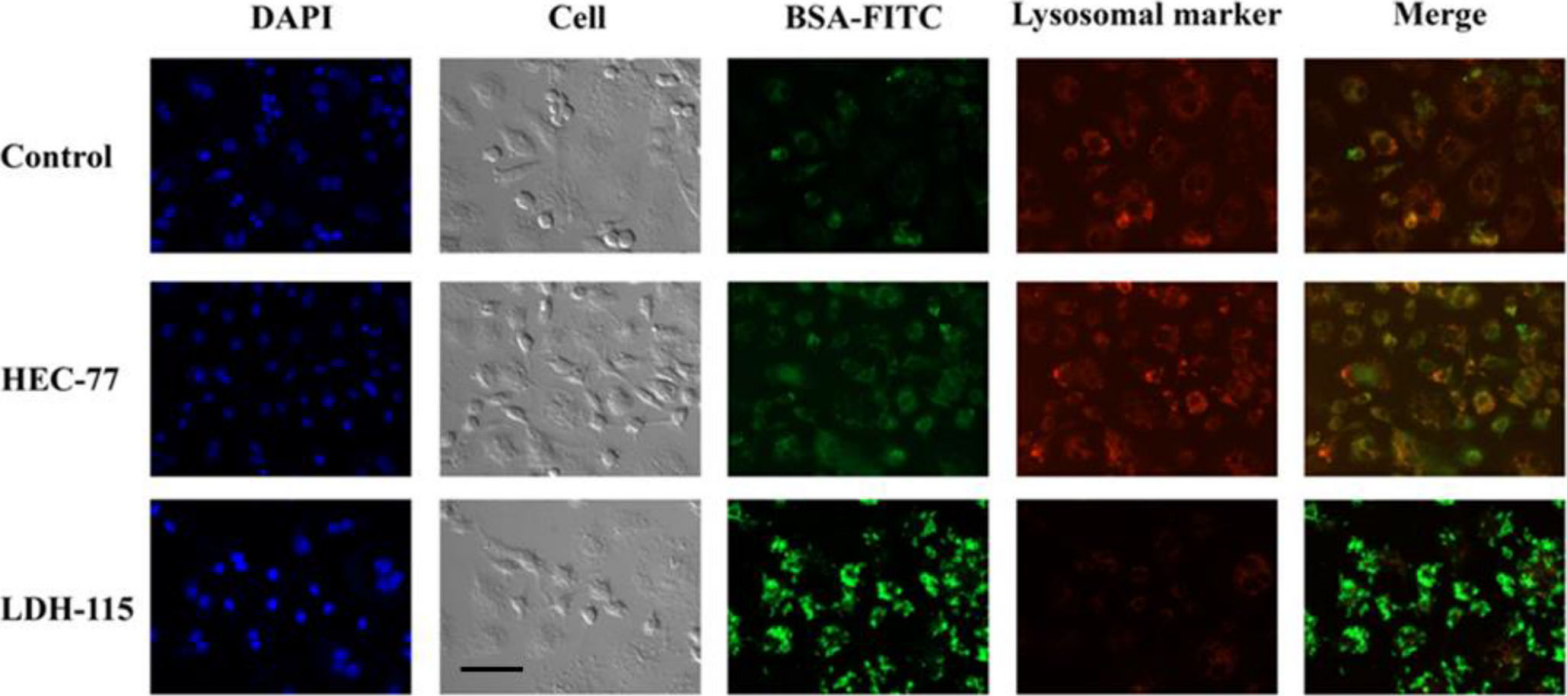
Antigen delivery mediated by LDH and enhanced protein cellular uptake. RAW 264.7 cells were incubated for 1 h with BSA-FITC (control), BSA-FITC-HEC-77 (green) or BSA-FITC-LDH-115 (green) and Lysotracker (Red), then fixed and imaged. Scale bar = 50 μm. Reproduced with permission (11). Copyright 2018 Wiley-VCH.

The endosome escape has been confirmed by monitoring the cellular uptake process using the lysosomal marker. As shown in Fig. 4, BSA-FITC and HEC-77-BSA-FITC (‘77’ meant the size was 77 nm) were taken up by macrophage cells. In these two cases, clear lysosomal marker (red fluorescence) overlapping with the green BSA-FITC fluorescence was observed, revealing that their endocytosis both undergoes the lysosome pathway. In sharp contrast, there was not any red fluorescence observed in macrophages treated with LDH-115-BSA-FITC (‘115’ meant the size was 115 nm). Instead, very strong green fluorescence was observed, meaning that LDH particle only undergoes the endosome escape pathway (11). Moreover, LDH-115 delivered much more BSA-FITC into the cells (Fig. 4) than HEC and BSA-FITC itself, in accordance with the quick cytoplasmic delivery via the endosome pathway. Therefore, delivery of BSA (or protein in general) individually or complexed with HEC nanoparticles undergoes the lysosomal escape, while BSA delivery via LDH nanoparticles is achieved through the endosomal escape in a more efficient way.

In addition, the endosome escape has been also reported for LDH internalization by HEK 293 (Human embryonic kidney 293) cells (36) and HOS cells (cancer cell lines) (37).

### Calcium Phosphate (CaP)

Calcium phosphate (CaP) nanoparticles are widely examined as an ideal delivery carrier, specifically for delivery of gene-based therapeutics for over 30 years (38). There are several types of CaP nanoparticles with different Ca/P molar ratios (39). This review just talks about CaHPO_4_ as a simple example, which is formed as precipitated nanoparticles in pH 6–9 buffers. CaP nanoparticles are often developed for plasmid DNA delivery (38, 40). Clearly, CaP-RNA/DNA nanomaterials were taken up by cells via the endosomal escape (41).

However, CaP nanoscale precipitates suffer from poor dispensability and colloidal instability in water caused by particle aggregation and crystalline phase changing (42, 43). In the last decade, various approaches have been developed to modify CaP particles to overcome these issues and enhance the endosome escape for more efficient delivery. For example, Li *et al*. developed lipid-coated calcium phosphate (LCP) nanoparticles for targeted gene and drug delivery, with very successful *in vivo* delivery for efficient cancer treatment (13, 44). Subsequently, Tang *et al*. developed a dual-targeted LCP nanoparticle system to efficiently accumulate LCP nanoparticles in the tumor tissue and effectively inhibited the tumor growth via combined genotherapy and phototherapy (45). Recently, Sun *et al*. employed alendronate and bovine serum albumin (BSA) to coat CaP nanoparticles to maintain the colloidal stability for efficient DNA vaccine delivery for cancer immunotherapy (46). Interestingly, mannose pre-conjugated onto some BSA specifically binds C-type lectin receptor on the antigen-presenting cell (APC) and increases the antigen presentation by APCs, leading to a higher anti-cancer immunity.

**Fig. 5:**
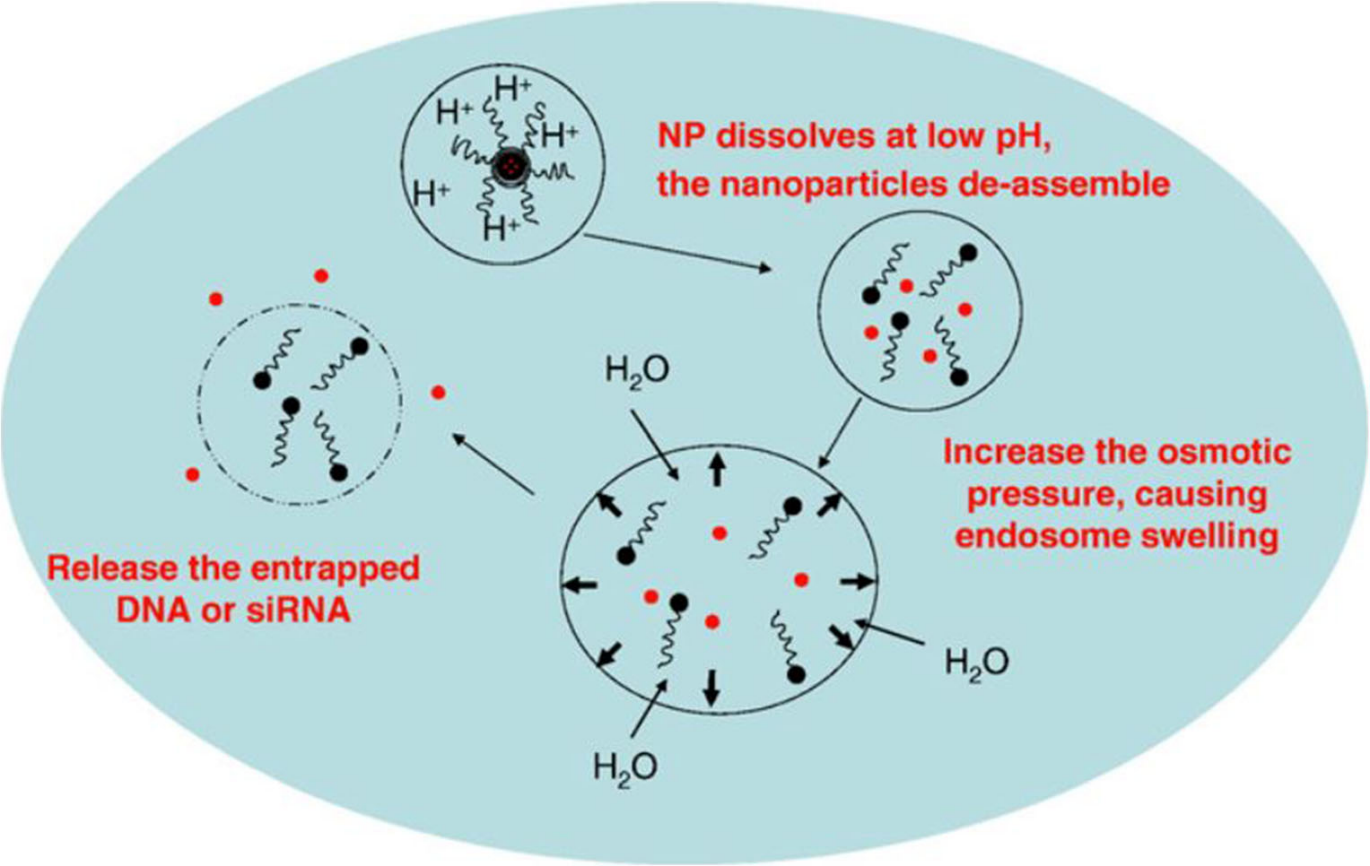
The hypothesized four steps for release of LCP-siRNA from the endosome. (1) The LCP enters the cell in the form of early endosome. (2) The CaP core is dissolved in acidic endosome for NP de-assembly. (3) The osmotic pressure due to dissolved calcium and phosphate ions causes endosome swelling. (4) The endosome bursts and releases all endosomal contents into cytoplasm. Reproduced with permission (13). Copyright 2010, Elsevier.

CaP particles are found to be endocytosed by various cells via the endosome escape and avoid the subsequent lysosomal degradation and/or exocytosis (41), which is important for gene delivery as the whole nucleic acid is basically required to deliver into the cytoplasm for the subsequent biofunction. Similar to LDH nanoparticles, poorly soluble calcium phosphate (supposed CaHPO_4_ precipitate) naturally dissolves in acidic conditions by consuming protons in the endosome (Fig. 5):

5 $$ \mathrm{CaHP}{\mathrm{O}}_4+{\mathrm{H}}^{+}\to {\mathrm{Ca}}^{2+}+{\mathrm{H}}_2\mathrm{P}{{\mathrm{O}}_4}^{-} $$

The major component of CaP particles is amorphous CaHPO_4_ (pKsp = 6.56, or 0.02 g/100 mL) as CaP precipitation occurs normally at pH around 7–8 where HPO_4_^2−^ is the major phosphate species. The precipitate (CaHPO_4_) can thus be dissolved in the acidic condition because protonated product Ca(H_2_PO_4_) is much more soluble (2.0 g/100 mL). The proton depleting property of CaP particles has been confirmed by acid-base titration assays, which causes the increase of salt ion concentration, disruption of the endosome and releases of CaP-therapeutic into the cytoplasm. Thus, CaP-therapeutic nanoparticles are successfully delivered into the cytoplasm before enzyme-degradation in the lysosome (Fig. 5). The acidity-triggered dissolution of CaP precipitates and the subsequent endosomal escape significantly improved the delivery of siRNA (14, 45, 47), which was further enhanced when CaP nanoparticles were modified with charge-conversional polymer (48).

### Calcium Carbonate (CaC)

Calcium carbonate (CaC) nanoparticles are also examined as the delivery system (49), as they have some advantages, such as high biocompatibility, low toxicity, sensitive pH-responsiveness (50), easy availability, biodegradability and high capacity for carrying different groups of drugs. CaC nanoparticles are often used as release-controllable delivery systems for cancer therapy (49). CaC nanoparticles respond to the weak acidity and release therapeutics in a sustained manner as well. Specifically, two neutralization reactions take place in the endosome when they are internalized by tumor cells:

6 $$ \mathrm{CaC}{\mathrm{O}}_3+{\mathrm{H}}^{+}\to {\mathrm{Ca}}^{2+}+\mathrm{HC}{{\mathrm{O}}_3}^{-} $$

7 $$ \mathrm{HC}{{\mathrm{O}}_3}^{-}+{\mathrm{H}}^{+}\to {\mathrm{H}}_2\mathrm{O}+\mathrm{C}{\mathrm{O}}_2 $$

**Fig. 6:**
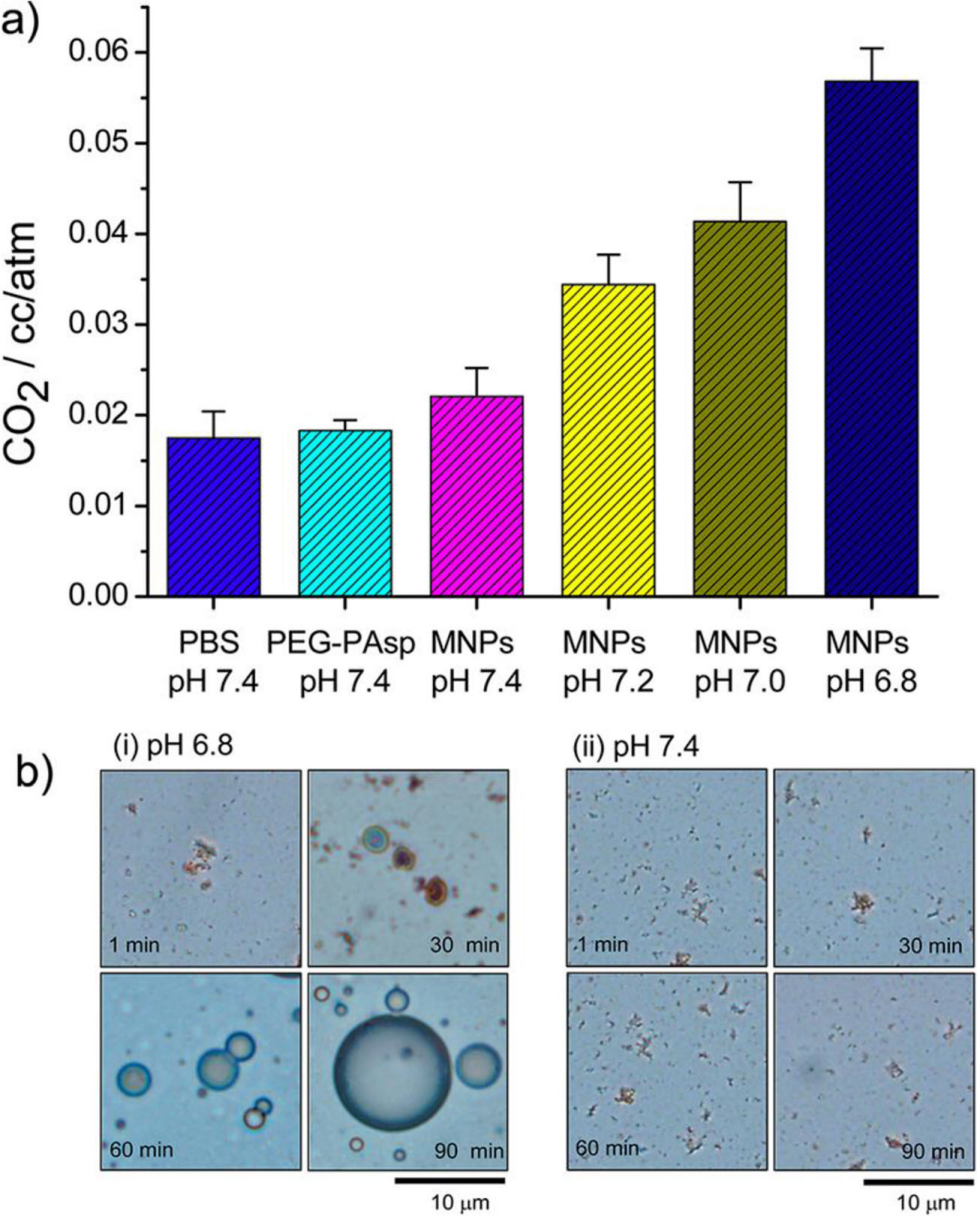
(**a**) Amount of CO_2_ generated from DOX-CaCO_3_-MNPs at various pH values and (**b**) optical micrographs of CO_2_ bubbles generated by incubating DOX-CaCO_3_-MNPs in PBS ((i) pH 6.8 and (ii) pH 7.4) for 90 min. Reproduced with permission (21). Copyright 2015 American Chemistry Society.

The dissolution reactions start even at pH 7.2–7.4 (21). As shown in Fig. 6, CaC nanoparticles started to dissolve and generated CO_2_ at pH 7.4. The generation of CO_2_ seemed much quicker in buffers with the pH level decreasing from 7.2, 7.0, to 6.8. The CO_2_ generation from CaC nanoparticles can be attributed to the inherent pH-sensitive property as protonated Ca(HCO_3_)_2_ is very highly soluble (16.6 g/100 mL) and continuous acidification generates CO_2_ to consume H^+^ ions and bicarbonate ions.

The increased concentration of Ca^2+^ and Cl^−^ accompanying the pumped H^+^ ions in the endosome causes a high osmotic pressure and influx of H_2_O to expand the endosome. Together with the generation of gaseous CO_2_, the endosome is finally burst out, releasing CaC-therapeutic nanoparticles into the cytoplasm. This is also called as ‘CO_2_ blast effect’ specifically for carbonate nanomaterials (22). In addition, the generated CO_2_ bubbles can be further used as a contrast agent for ultrasound imaging of the tumor microenvironment (21).

**Fig. 7:**
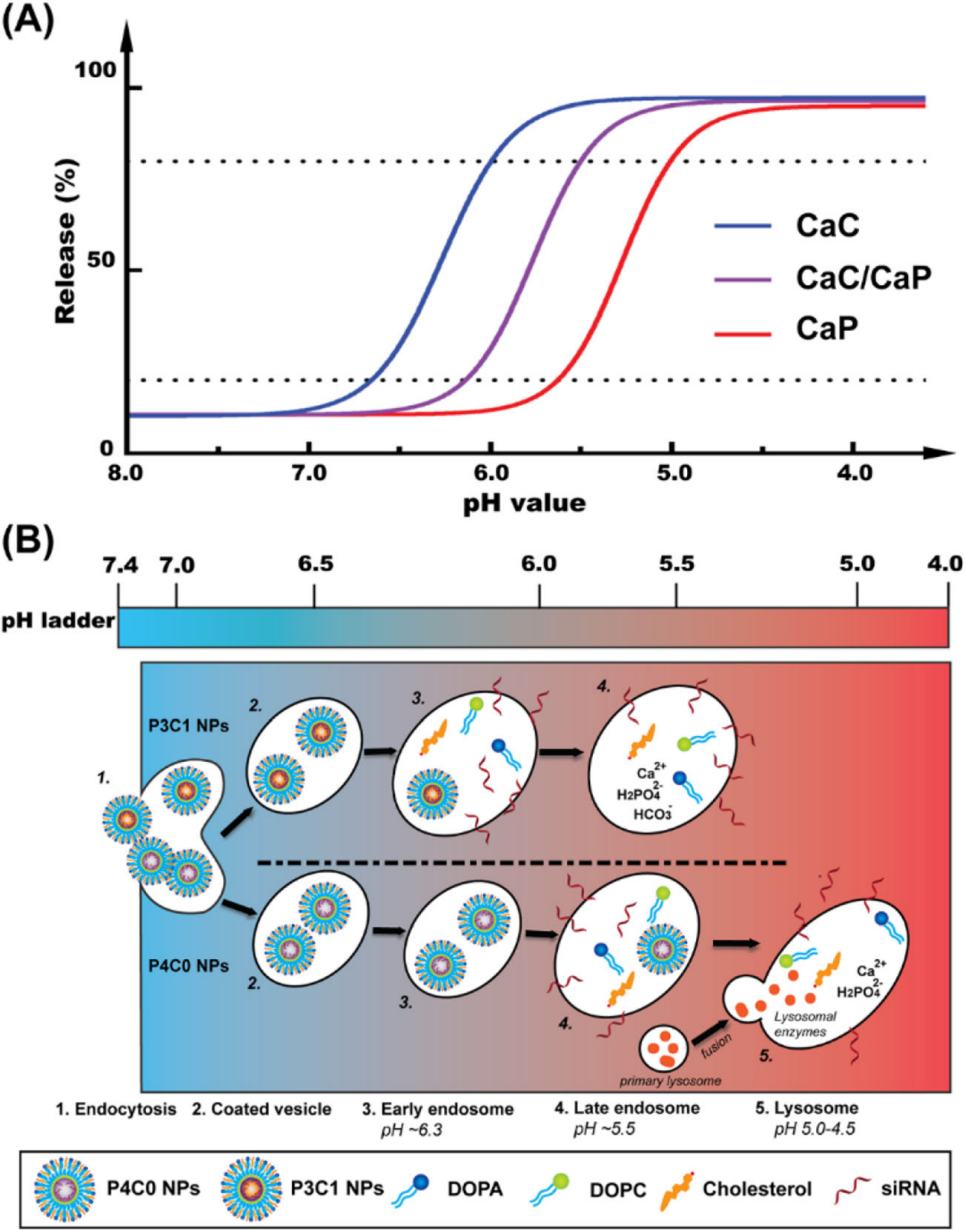
(**A**) Schematic pH responsive profiles of CaC/CaP hybrid nanoparticles. The hybrid CaC/CaP nanoparticles specifically dissolve and escape within pH 6.0–5.5; (**B**) The possible processes of CaP (P4C0) and CaC/CaP (P3C1) nanoparticles with the phosphate/carbonate molar ratio of 4:0 and 3:1, respectively, during internalization. The clathrin-mediated endocytosis undergoes the endosome/lysosome digestion and escape pathway. The pH value dropped from 7.4 in Step 1 to below 5.0 in Step 5 (lysosome). CaC/CaP (P3C1) and CaP (P4C0) nanoparticles may start dissolution in Step 3 and Step 4 and then escape in Step 4 and Step 5, respectively. Reproduced with permission (51). Copyright 2017 The Royal Society of Chemistry.

Since CaC nanoparticles start to dissolve in pH 7.4 buffers, the carried therapeutics may be released and lost in the blood circulation to cause some side effects. Moreover, in the tumor extracellular environment (pH 6.5–6.9), more therapeutics may be released and lost before cellular uptake, which may reduce the delivery efficacy to tumor cells. To overcome this early release issue, we have recently developed lipid coated calcium phosphate/carbonate (LCCP) hybrid nanoparticles (51). This calcium phosphate/carbonate system does not release the cargo during the blood circulation, while helps achieve the early/late endosome escape and completely avoid the lysosomal pathway. As schematically outlined in Fig. 7, CaP nanoparticles (P4C0, phosphate/carbonate molar ratio of 4:0) may escape in the late endosome and/or lysosome (pH 5.0–5.5), as some lysosome trackers are reported to colocalize with CaP nanoparticles (52). To the contrary, CaC nanoparticles are sensitive to the neutral pH (21), and probably escape from the early endosome (pH 6.0–6.5). For the hybrid CaP/CaC (P3C1, phosphate/carbonate molar ratio of 3:1) nanoparticles, the dissolution mainly takes place in pH 5.5–6.0, exactly leading to the endosome escape and perfectly protecting the bioactive by reducing the leaking in neutral pH and avoiding the possible lysosomal degradation (51).

### Miscellaneous

There are several other inorganic nanomaterials that can be potentially developed as biocompatible delivery systems with the endosome escapability, including Mg(OH)_2_, MgO, MgCO_3_, Zn(OH)_2_, and Zn_5_(OH)_8_(NO_3_)_2_ (53). The common feature of these biocompatible inorganic nanoparticles is weak alkalinity, which endows these nanomaterials high pH-sensitivity in the weakly acidic environment (such as the endosome) and helps undergo the endosome escape for the endoplasmic delivery of the intact bioactives loaded in these nanoparticles.

## Summary and Prospect

As reviewed in this paper, there are several inorganic nanomaterials that can be developed as delivery vehicles and naturally help nanoparticle-bioactive hybrids escape from the endosome and safely deliver to the cytoplasm. The review paper has discussed a few such inorganic nanomaterials, e.g. LDH, CaP, CaC and some hydroxides. These nanomaterials possess two common yet distinguished properties:

1. Reasonable sensitivity to the weak acidity (pH 5–7) and inherent neutralization with H^+^ ions via spontaneous dissolution reactions.
2. Poor solubility in neutral solutions (pH 7.4) but enhanced solubility in acidic solutions (pH 5–6).

These two properties enable the nanomaterials to partially dissolve and increase the ion strength in the endosome, which generates the osmotic pressure and causes more water from the cytosol to enter, swell and finally burst the endosome, achieving the successful cytoplasmic delivery via the endosome escape.

Based on the summary, the author would foresee that there would be more investigations in order to develop inorganic nanomaterial-based delivery systems for more efficient delivery of bioactives via the endosomal escape pathway. These inorganic nanomaterial-based systems may involve:

1. new weakly alkaline inorganic nanomaterials (M_x_A_y_) will be investigated as functional delivery systems, including those with M = Li^+^, Zn^2+^, Mg^2+^, Ca^2+^, Sr^2+^, Ba^2+^ and A = O^2−^, OH^−^, CO_3_^2−^, and (H_m_PO_4_)^m-3^ (m = 0, 1, or 2); There are several nanomaterials already investigated, such as ZnO, CaP, CaCO_3_, and MgCO_3_.
2. novel mixed inorganic nanomaterials (M1_x1_M2_x2_A1_y1_A2_y2_), where M1, M2 may be one of above cations and A1 and A2 one of the above anionic groups; LDH, CaP/CaC, Zn_5_(OH)_8_(NO_3_)_2_, and Zn_5_(OH)_8_(CO_3_) are examples.
3. more hybrid inorganic nanomaterials (M_x_A_y_@(bio)organic molecules).

In particular for item 3, biomolecules (such as CPPs) and polymers (charge conversional PEG) can be hybridized with both normal inorganic nanomaterials (such as Fe_3_O_4_, Au, Ag, carbon nanomaterials etc) and weakly alkaline inorganic nanomaterials (M_x_A_y_ and M1_x1_M2_x2_A1_y1_A2_y2_) to achieve endosome escape for efficient cytoplasmic delivery, which may well enrich the strategy for developing hybrid nanoparticles as more efficient cytoplastic delivery of bioactives.

## ABBREVIATIONS

ATPase: Adenosine 5′-triphosphatase
AuNP: Gold nanoparticle
BSA: Bovine serum albumin
CaC: Calcium carbonate
CaP: Calcium phosphate
CHO: Chinese hamster ovary cells
COS: Cells being **C**V-1 (simian) in **O**rigin, and carrying the **S**V40 genetic material
CPPs: Cell penetration peptides
DOX: Doxorubicin
FITC: Fluorescein isothiocyanate
HEC: Hectorite
HEK 293: Human embryonic kidney 293 cells
HOS: cancer cell line name
IONP: Iron oxide nanoparticle
LCCP: Lipid-coated calcium phosphate/carbonate
LCP: lipid-coated calcium phosphate
LDH: Layered double hydroxide
MNPs: Mineralized nanoparticles
PBS: Phosphate-buffered saline
PEI: Polyethyleneimine
TAT: Transactivator of transcription
TEM: Transmission electron microscope
